# Fusariosis en pacientes con cáncer: serie de 13 casos y revisión de la literatura

**DOI:** 10.7705/biomedica.6925

**Published:** 2023-08-31

**Authors:** Sonia Isabel Cuervo-Maldonado, José Camilo Álvarez-Rodríguez, Cristian Leonardo Cubides, Juan Camilo Barrera, Juan Diego Montañez-Abril, Erika Paola Vergara-Vela, Carlos Humberto Saavedra-Trujillo, María José López-Mora, Gloria Elena Mora-Figueroa, Adriana Celis-Ramírez, Rose Mary Jaramillo-Calle, Rafael Parra-Medina

**Affiliations:** 1 Facultad de Medicina, Universidad Nacional de Colombia, Bogotá, D.C., Colombia Universidad Nacional de Colombia Facultad de Medicina Universidad Nacional de Colombia Bogotá D.C Colombia; 2 Grupo de Infectología, Instituto Nacional de Cancerología, Bogotá, D.C., Colombia Grupo de Infectología Instituto Nacional de Cancerología Bogotá D.C Colombia; 3 Grupo de Investigación en Enfermedades Infecciosas en Cáncer y Alteraciones Hematológicas (GREICAH), Facultad de Medicina, Universidad Nacional de Colombia, Bogotá, D.C., Colombia Universidad Nacional de Colombia Grupo de Investigación en Enfermedades Infecciosas en Cáncer y Alteraciones Hematológicas (GREICAH) Facultad de Medicina Universidad Nacional de Colombia Bogotá D. Colombia; 4 Grupo de Enfermedades Infecciosas, Facultad de Medicina, Universidad Nacional de Colombia, Bogotá, D.C., Colombia Universidad Nacional de Colombia Grupo de Enfermedades Infecciosas Facultad de Medicina Universidad Nacional de Colombia Bogotá D.C Colombia; 5 Grupo de Infectología, Clínica de Marly, Bogotá, D.C., Colombia Grupo de Infectología Clínica de Marly Bogotá D.C Colombia; 6 Grupo de Investigación Celular y Molecular de Microorganismos Patógenos (CeMoP), Universidad de los Andes, Bogotá, D.C., Colombia Universidad de los Andes Grupo de Investigación Celular y Molecular de Microorganismos Patógenos (CeMoP) Universidad de los Andes Bogotá D.C Colombia; 7 Grupo de Investigación Patología Oncológica INC, Instituto Nacional de Cancerología, Bogotá, D.C., Colombia Grupo de Investigación Patología Oncológica INC Instituto Nacional de Cancerología Bogotá D.C Colombia

**Keywords:** Fusarium, fungemia, anfotericina B, voriconazol, espectrometría de masa por láser de matriz asistida de ionización desorción, Fusarium, fungemia, amphotericin B, voriconazole, spectrometry, mass, matrix-assisted laser desorption-ionization

## Abstract

La fusariosis es una micosis oportunista producida por *Fusarium* spp. Su presentación clínica depende del estado inmunológico del huésped, especialmente, el de aquellos con enfermedades hematooncológicas, cuyas manifestaciones varían desde formas localizadas hasta infección fúngica invasora. El cultivo de piel o de sangre permite orientar el tratamiento antifúngico combinado con anfotericina B y voriconazol. Se presentan 13 casos de pacientes con cáncer en un periodo de once años que desarrollaron fusariosis diseminada; asimismo, se hizo con una revisión extensa de la literatura. En esta serie de casos, la mortalidad fue del 61,5 % (8/13), a pesar del uso del antifúngico. De los 13 pacientes, 11 tenían neoplasia hematológica y 2 neoplasia sólida. El factor de riesgo más importante fue la neutropenia profunda. El compromiso de la piel y los hemocultivos positivos facilitaron la prescripción del tratamiento combinado en la mayoría de los casos. La neutropenia febril persistente asociada a lesiones cutáneas, la onicomicosis, los nódulos o las masas pulmonares permitieron sospechar una infección fúngica invasora por *Fusarium* spp.

El objetivo de la presentación de esta serie de casos es recordar el diagnóstico de fusariosis a la comunidad médica en contacto con pacientes oncológicos, con neutropenia febril profunda y persistentes.

## Abstract (en)

The fusariosis is an opportunistic mycosis caused by *Fusarium* spp. Its clinical presentation depends on the immunological status of the host, especially in patients with hemato-oncological diseases, whose manifestations vary from localized to invasive fungal infections. Skin or blood culture helps to guide combined antifungal treatment with amphotericin B and voriconazole.

Here, we present 13 cases in a period of eleven years of patients with cancer who developed disseminated fusariosis and their outcomes, together with a review of the related literature. In this series of cases, mortality was 61.5 % (8/13), despite the use of the antifungal. Out of the 13 cases, 11 had hematological neoplasia and 2 solid neoplasia. The most determinant risk factor was profound neutropenia. Skin involvement and positive blood cultures in most cases allowed combined treatment prescription. Persistent febrile neutropenia associated with skin lesions, onychomycosis, nodules, or lung masses lead to suspicion of *Fusarium* spp. fungal invasive infection.

The aim of this series of cases is to remind healthcare professionals that oncological patients with deep and persistent febrile neutropenia can develop fusariosis.

*Fusarium* spp. es un hongo ubicuo en la naturaleza, filamentoso, hialino y tabicado que hace parte de las hialohifomicosis y produce enfermedad en las plantas, los animales y el hombre.

La infección puede ser localizada o diseminada (infección fúngica invasora). Esta última es la forma predominante en pacientes inmunocomprometidos como aquellos con neoplasias hematológicas, leucemias agudas, o que han sido sometidos a trasplante de progenitores hematopoyéticos o de órgano sólido. En estos pacientes, la mortalidad puede alcanzar el 45 %. Una neutropenia grave -con menos de 100 células por con una duración mayor de siete días, altas dosis de corticoides y linfopenia se han identificado como factores de riesgo para el desarrollo de fusariosis.

En Colombia, la infección fúngica invasora se ha informado esporádicamente como reporte de caso, sin que a la fecha y en conocimiento de los autores se disponga de una revisión sistemática o de un informe de serie de casos que permita identificar sus particularidades.

Esta serie de casos presenta las características epidemiológicas, clínicas, diagnósticas y terapéuticas en pacientes con cáncer para orientar a la comunidad médica, que trata este grupo de pacientes, sobre la búsqueda activa de la infección fúngica invasora por *Fusarium.*

## Casos clínicos

De la base de datos de un estudio de investigación sobre fungemia en pacientes con neoplasias hematológicas en instituciones de Bogotá, desarrollado entre el 2012 y el 2019 (código C19010300-473), y con la información del sistema de vigilancia activa institucional desde el 2020 en adelante, se identificaron 13 pacientes con cáncer y diagnóstico de fusariosis entre el 2012 hasta el 2022. La investigación fue aprobada por los comités de ética de las instituciones participantes y debido a las características del estudio retrospectivo no se necesitó de consentimiento informado.

Las características demográficas, clínicas, micológicas y terapéuticas, y los desenlaces se presentan en el cuadro 1.

**Cuadro 1:** Descripción de casos clínicos de pacientes con infección por *Fusarium* spp.

	Edad	Sexo	Neoplasia	Estado del cáncer	Esquema de quimioterapia	Recuento absoluto de neutrófilos	Especie	Cultivo	Coinfección	Profilaxis antifúngica	Tratamiento	Galactomanano	Desenlace
1	53+	M	LMA	Progresión	7+3	10	*Fusarium* spp.	Piel y sangre (hemocultivo positivo a los 3 días, 5 horas y 4 minutos de incubación)	*Corynebacterium* *striatum*	Posaconazol (previo al ingreso y durante 26 días intrahospitalarios)	Anf B liposómica (16 días) + VCZ (6 días); Al egreso con VCZ	0,062	Vivo
2	27++	H	Linfoma/LLA	Recaída	HyperCVAD	0	*Fusarium* *solani*	Piel y sangre (hemocultivos 2/2 positivos a las 44,15 horas y a las 32,14 horas de incubación)	*Corynebacterium* *jeikeium*	Fluconazol (al inicio de la quimioterapia)	Anf B (14 días) + VCZ (10 días)	No	Fallece
3	21	H	LLA PreB	Resistente al tratamiento	GRAALL	20	*Fusarium* spp.	Piel y sangre	*Enterococcus* *faecium*		Anf B liposómica + VCZ	0,051	Fallece
4	25	H	Tumor germinal no seminomatoso	Progresión	PosTPH hematopoyéticos autólogo, preinjerto (día +8)	30	*Fusarium* spp.	Piel y sangre	*Escherichia coli*		Anf B liposómica +VCZ	No	Fallece
5	33	M	Linfoma Hodgkin clásico, tipo esclerosis nodular	Postrasplante preinjerto (día +14)	TPH alogénico	30	*Fusarium* *oxysporum*	Sangre a través de catéter y periférica (hemocultivos positivos a las 49,4 horas y a las 85,21 horas respectivamente)	No documentadas	Fluconazol (al inicio de la quimioterapia)	Anf B liposómica (18 días) + VCZ (32 días); al egreso con VCZ	0,068	Vivo
6	54	H	LMA	Tercera recaída	Postrasplante de progenitores hematopoyéticos alogénico haploidéntico, pre-injerto (día+19)	1	*Fusarium* spp.	Piel y sangre	No documentadas		Anf B liposómica +VCZ	No	Fallece
7	49*	H	Linfoma NK extraganglionar	Recaída	TPH alogénico en recaída - GDP	0	*Fusarium* *solan*	Sangre y SPN (hemocultivo positivo a los 71,25 horas)	*Klebsiella* *pneumoniae* productora de carbapenemasa	Voriconazol 100 Anf B (8 días) (previo al ingreso y durante la quimioterapia)	mg/cada 12 horas +VCZ (8 días a dosis de 200 mg/12 horas)	No	Fallece
8	17	H	LLA PreB	Resistente al tratamiento	ALL-R3	50	*Fusarium* spp.	Piel y sangre	No documentadas		Anf B +VCZ	0,053	Fallece
9	41	M	LMA (M3)	Recaída	7+3	20	*Fusarium* spp.	Piel y sangre	Actinomyces		VCZ	No	Fallece
10	64	M	Sarcoma fusocelular de alto grado retroperitoneal	Progresión	Ninguno	15322	*Fusarium* *solan*	Sangre (hemocultivo positivo a las 78,16 horas	No documentadas		Ninguno (diagnóstico post mortem)	No	Fallece
11	49	H	LLA	Progresión	Hyper CVAD	30	*Fusarium* spp.	Orina (Sin reporte de tiempo de positividad)	No documentadas		VCZ (7 días intrahospitalario)	No	Vivo
12	44	M	LLA PreB	Progresión	IDA FLAG	20	*Fusarium* spp.	Sangre (hemocultivos positivos a los 84,3 horas y a las 96,89 horas)	No documentadas	Fluconazol (previo al inicio de la quimioterapia)	Anf B (24 días) + VCZ (27 días)	No	Vivo
13	19	M	LLA PreB	Recaída	IDA FLAG	18	*Fusarium* spp.	Sangre (hemocultivos positivos a los 101,45 horas y a las 90,40 horas)	No documentadas	Fluconazol (previo al inicio de la quimioterapia)	VCZ (34 días)	1,3	Vivo

LMA: leucemia mieloide aguda; LLA: leucemia linfoblástica aguda; TPH: trasplante de progenitores hematopoyéticos; SPN: senos paranasales; Anf B: anfotericina B; VCZ: voriconazol

+ Pacientes a quienes se les realizó biopsia de piel

++ Descripción de la biopsia de piel correspondiente a la figura.

De los 13 casos, 6 eran mujeres, la mediana de edad fue 41 años; 11 tenían diagnóstico de neoplasia hematológica: 8 con leucemia aguda y 3 con linfoma; solo 2 pacientes tenían cáncer sólido: 1 con tumor germinal no seminomatoso y antecedentes de trasplante de progenitores hematopoyéticos autólogo y 1 con sarcoma fusocelular retroperitoneal de alto grado. Doce pacientes estaban en tratamiento activo del cáncer con quimioterapia antes del diagnóstico de fusariosis y cuatro estaban en diferentes tiempos posterior al trasplante de progenitores hematopoyéticos. La mediana del recuento absoluto de neutrófilos fue de 20 células por µl en el momento del diagnóstico de la fusariosis, con excepción de un paciente con sarcoma fusocelular retroperitoneal, que presentó un recuento absoluto de neutrófilos de 15.322 células por µl.

**Figura 1:**
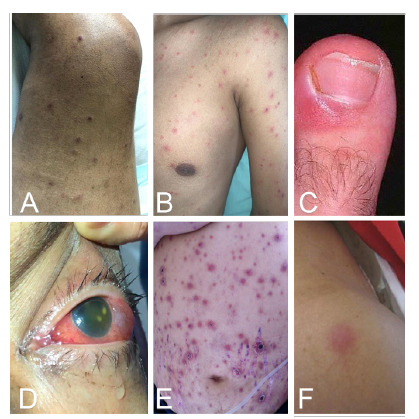
Hallazgos clínicos de los pacientes con manifestaciones cutáneas u oftálmicas por *Fusarium* spp. **A.** Lesiones nodulares eritematosas, algunas en fase costrosa con compromiso de miembros inferiores (caso 8). B. Lesiones pápulo-nodulares diseminadas en cabeza, tronco y extremidades (caso 2). **C.** Paroniquia del dedo gordo del pie izquierdo (caso 4). **D.** Endoftalmitis aguda unilateral (caso 6) **E.** Máculas, pápulas y placas eritematosas en toda la región abdominal con distribución aleatoria, algunas con costra necrótica central. **F.** Lesión nodular eritematosa en el hombro izquierdo (caso 4).

Entre las características clínicas para el diagnóstico de fusariosis, se encontró el compromiso de la piel, con lesiones tipo mácula, pápula o nódulos eritematosos de distribución aleatoria en 7 de los 13 casos y uno de los pacientes presentó compromiso oftálmico (endoftalmitis) (figura 1 A-1F). La prueba de galactomanano sérico -presente en la pared celular fúngica- se practicó en 5 de los 13 pacientes y sólo uno tuvo un valor de galactomanano de 1,3 ng/ml (considerado elevado) en el momento del diagnóstico de fusariosis.

**Figura 2:**
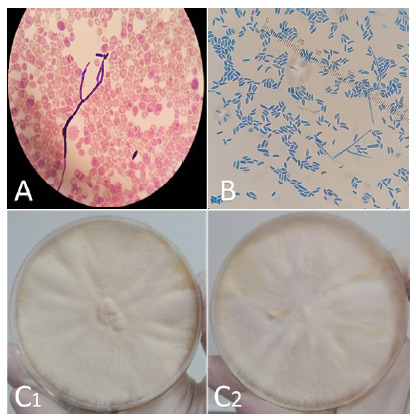
Diagnóstico micológico: **A.** Coloración de Gram de un hemocultivo, 100X. Se observa hifa septada o tabicada y macroconidias en forma de banana. **B.** Examen directo de cultivo con coloración de azul de lactofenol, 400X. Se observan macroconidias de *Fusarium* spp. en hemocultivo. **C.** Apariencia de las colonias crecidas en agar Sabouraud (anverso). C1. Cultivo de *Fusarium solani;* C2. Cultivo de *Fusarium oxysporum.*

En 12 casos, la identificación de *Fusarium* spp. se hizo a partir de hemocultivos (figura 2A) y en 6 de hemocultivos y piel; en 6 pacientes se documentó coinfección bacteriana, dos con *Corynebacterium* spp., uno con *Actinomyces* spp., otro con *Enterococcus faecium*y dos con *Enterobacterales.*

**Figura 3:**
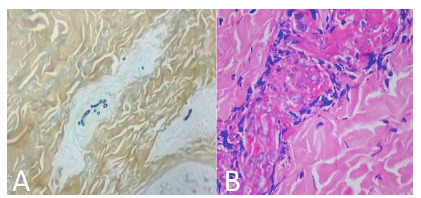
Hallazgos histopatológicos en biopsia de piel: **A.** Se resalta la presencia de escasas estructuras micóticas en forma de hifas ramificadas en las luces vasculares. Gomori, 10X. Los hallazgos histopatológicos sugieren infección por *Fusarium* spp. como primera posibilidad. **B.** Se observa piel revestida por epidermis de espesor usual, dermis con mínimo infiltrado inflamatorio mononuclear, sin atipia ni otros hallazgos relevantes. Hematoxilina y eosina, 20X.

**Figura 4:**
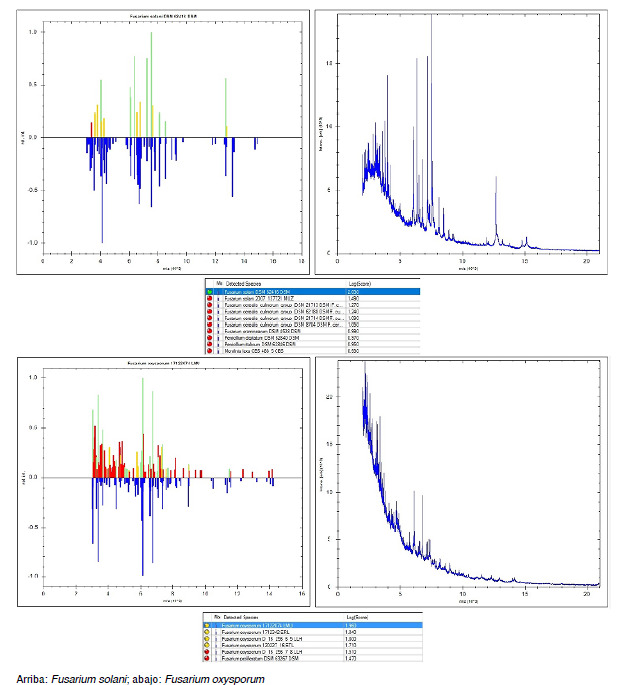
Identificación de especies de *Fusarium* mediante espectrometría de masas de desorción-ionización láser, asistida por matriz (MALDI-TOF).

En tres casos se obtuvo biopsia de piel (figura 3A y 3B). Los hallazgos de la biopsia, teñida con hematoxilina y eosina, mostraron piel revestida por epidermis de espesor usual, dermis con mínimo infiltrado inflamatorio mononuclear sin atipia, ni otros hallazgos relevantes. Las coloraciones con ácido peryódico de Schiff y la de Gomori resaltaron escasas estructuras micóticas en forma de hifas ramificadas en las luces vasculares. Los hallazgos histopatológicos sugirieron infección por *Fusarium* spp. como primera posibilidad. En cuatro aislamientos, mediante la espectrometría de masa de desorción-ionización láser, asistida por matriz (MALDI-TOF) (figura 4), se identificó la especie: en tres casos fue *Fusarium solani* y en uno, *F. oxysporum;* los nueve restantes se clasificaron como *Fusarium* spp.

En nueve casos, el tratamiento antifúngico fue una combinación de anfotericina B y voriconazol; tres pacientes recibieron monoterapia con voriconazol y uno no alcanzó a recibir tratamiento porque el diagnóstico fue *post mortem.* Finalmente, solo en cinco pacientes la supervivenca superó los 28 días.

### Epidemiología

La fusariosis invasiva es una enfermedad infrecuente ^1^ que afecta niños y adultos ^2^. Está asociada a neoplasia hematológica en el 87 % de los casos ^1^. Aunque es un hongo ubicuo en la naturaleza, la prevalencia de la infección invasiva es bastante baja, estimada entre el 0,06 y el 0,13 % en pacientes con neoplasias hematológicas ^3^. También se ha asociado a trasplante de órgano sólido con una incidencia de 5,97 casos por 1.000 trasplantes de órgano sólido ^2^. En el *MD Anderson Cancer Center,* entre 1998 y 2009, se encontraron 44 casos de fusariosis invasiva: el 84 % se presentó en pacientes con leucemias agudas o síndromes mielodisplásicos y el 16 % en pacientes con leucemias crónicas o linfomas ^4^. Del total, el 48 % fue sometido a trasplante de progenitores hematopoyéticos y el 52 % a dosis altas de esteroides en los 30 días anteriores al diagnóstico ^4^, datos similares a los encontrados en esta serie de casos.

La prevalencia de fusariosis en pacientes con trasplante de progenitores hematopoyéticos y leucemia mieloide aguda en Estados Unidos y Europa es menor del 1 %, mientras que en Sudamérica es del 5 % en pacientes con trasplante de progenitores hematopoyéticos ^5^. La especie varía por región; por ejemplo, el complejo de especies *Fusarium fujikuroi* es predominante en Europa ^6^, mientras que el complejo de especies *Fusarium solani* es común en Brasil ^7^. En Colombia, a la fecha no se dispone de información al respecto, los casos clínicos descritos se informan como *Fusarium* spp ^8^ y solo en un caso se identificó la especie *F. verticilloides*^9^.

### Etiología y factores de riesgo

Las especies de *Fusarium* son mohos ubicuos ^4^, filamentosos, tabicados, que se bifurcan en ángulo agudo; son comunes en el suelo ^10^ y el agua ^2^. El género *Fusarium* está comprendido por 78 especies y 10 complejos de especies. Los complejos más frecuentes que causan infección en humanos son: *F. solani, F. oxysporum, F. incarnatum-equiseti, F. chlamydosporum, F. tricinctum, F. dimerum*y *F. fujikuroi*^6^^,^^11^.

Las especies de *Fusarium* causantes de infecciones invasivas y diseminadas en humanos son del complejo *Fusarium solani* (50 % de los casos notificados) ^3^^,^^8^, *Fusarium oxysporum* (20 %) y *Fusarium verticillioides,* que forma parte del complejo *Fusarium fujikuroi* (responsable del 20 % de las infecciones).

De los once años en los que se recopiló la serie de casos, solo en los últimos cuatro las instituciones participantes dispusieron de MALDI-TOF con la que lograron la identificación de especies en cuatro casos: las especies *F. solani* y *F. oxysporum* (figura 4) fueron las más frecuentes y causantes de infección fúngica invasora, lo cual concuerda con lo descrito en la literatura.

Las especies de *Fusarium* poseen varios factores de virulencia, como la producción de micotoxinas capaces de suprimir la inmunidad humoral y celular, para causar descomposición de los tejidos y también para producir proteasas y colagenasas ^12^.

Otros factores de riesgo reportados en la literatura evidencian la presencia de neutropenia profunda y prolongada mucho más frecuente en pacientes con neoplasias hematológicas o en quienes reciben esquemas de quimioterapia que causan aplasias, en pacientes con leucemia mieloide aguda, consumidores de cigarrillo (4/7) y receptores de trasplante alogénico de precursores hematopoyéticos. El uso de la globulina antitimocítica en el esquema de acondicionamiento se asoció significativamente al desarrollo de fusariosis (p<0,001) al igual que la hiperglucemia -con requerimiento terapéutico de insulina-, pero con la forma de fusariosis invasiva. La presencia de enfermedad injerto contra huésped, grado III o IV, también se asoció con mayor riesgo de infección fúngica invasora por *Fusarium* spp. en el periodo temprano después del trasplante ^13^.

### Patogénesis

El hongo ingresa principalmente por las vías respiratorias, pero también puede hacerlo por el tracto gastrointestinal, la piel o mediante dispositivos como catéteres venosos centrales ^10^. Cuando la vía de ingreso es pulmonar, los conidios se transforman en hifas, dando origen a la infección invasiva ^10^. Cuando la puerta de entrada es cutánea, la infección suele iniciar como paroniquia, onicomicosis o celulitis, desde donde se extiende por vía hematógena a sitios distantes de la piel y a los órganos internos, ocasionando infección invasiva ^10^. Esta última se caracteriza por el tropismo del hongo a los vasos sanguíneos, lo que lleva a isquemia e infarto de los tejidos afectados ^10^.

El compromiso de la inmunidad innata es un requisito importante para que se desarrolle la enfermedad invasora, ya que las hifas son destruidas por los macrófagos y los neutrófilos en condiciones normales ^14^, mediante mecanismos citotóxicos oxidativos ^10^. Se ha reportado que el 82 % de los pacientes con fusariosis presenta neutropenia (neutrófilos absolutos inferiores a 1.000 células por ml), y de estos, el 61 % tienen menos de 100 células por ml ^4^. La mediana de duración de la neutropenia antes del diagnóstico de fusariosis es de 15 días. Sin embargo, el rango va desde 0 hasta 71 días ^4^.

Otro fenómeno que resalta la importancia de la neutropenia en la patogénesis de la enfermedad es la relación entre la respuesta al tratamiento y la función de los granulocitos ^10^. Los linfocitos T también cumplen un papel en la infección por *Fusarium* spp., como queda evidenciado en los casos de fusariosis en pacientes inmunodeprimidos no neutropénicos ^14^. Campo y colaboradores encontraron linfopenia en el 93 % de sus pacientes, aunque con mayor prevalencia de la neutropenia ^4^. Aun así, la incidencia de fusariosis en pacientes con VIH es baja, excepto cuando hay enfermedad neoplásica asociada ^10^.

Los factores de riesgo más frecuentes, identificados en esta serie de casos, fueron la neoplasia hematológica y la neutropenia profunda (con un recuento absoluto de neutrófilos menor de 100 por µl). La puerta de entrada del hongo se pudo inferir en los pacientes que presentaron onicomicosis y en aquel que portaba un dispositivo vascular, a partir del cual se identificó la entidad fúngica. En ninguno de los pacientes se confirmó compromiso pulmonar o de otros órganos diferentes a la piel.

*Fusarium* spp. también puede causar infecciones en personas inmunocompetentes, pero en esos casos se presentan cuadros clínicos limitados a un órgano o sistema, como onicomicosis, queratitis o artritis ^10^. En pacientes inmunosuprimidos, las infecciones localizadas progresan rápidamente a infección invasiva diseminada ^10^.

### Manifestaciones clínicas

Las infecciones por *Fusarium* spp. tienen un espectro amplio de presentación ^2^ con manifestaciones que dependen del estado inmune del huésped y de las distintas vías de entrada: respiratoria, gastrointestinal o traumatismos en la piel (dispositivos vasculares) ^15^.

Puede producir infecciones localizadas y diseminadas ^2^. Las infecciones localizadas pueden corresponder a queratitis micóticas, otomicosis, onicomicosis y celulitis, aunque se han reportado incluso eumicetomas ^2^^,^^15^^,^^16^.

En la enfermedad diseminada, las lesiones cutáneas se presentan en el 73 % de los pacientes ^17^ y se caracterizan por ser pápulas o nódulos eritematosos, dolorosos, que evolucionan rápidamente a lesiones con necrosis central, semejante a lesiones en diana, debido a la invasión de la luz de los vasos sanguíneos por las hifas de *Fusarium* spp. Las lesiones se pueden localizar en cara, tronco o extremidades ^2^^,^^4^^,^^18^, y tienen como diagnóstico diferencial otras infecciones micóticas como por *Candida* spp., *Histoplasma* spp. y *Cryptococcus* spp.

Otras manifestaciones cutáneas de la enfermedad diseminada son las vesículas o ampollas hemorrágicas (3 % de los casos) ^19^ y los abscesos (7 % de los casos) ^4^. Las lesiones de tipo ectima se deben a trombosis e infarto causados por la invasión de la luz de los vasos sanguíneos por las hifas de *Fusarium.* Las lesiones aparecen rápidamente dentro de uno a cinco días, en varias etapas de evolución. En la figura 1 se muestran diferentes manifestaciones clínicas en la piel que algunos de los pacientes de esta serie presentaron y que por sus características semiológicas -nódulos subcutáneos, pápulas con centro necrótico, distribución generalizada sin compromiso de mucosa- y el hallazgo de onicomicosis en uno de los pacientes (figura 1C), fueron clave para la sospecha de infección fúngica invasora por *Fusarium* spp.

El compromiso pulmonar, o fungomas, se presenta en casos limitados. También puede manifestarse con consolidaciones, infiltrados intersticiales, nódulos, cavitaciones y de manera infrecuente, hemorragia alveolar difusa. ^2^^,^^8^. Sin embargo, en ninguno de los pacientes de la serie se confirmó compromiso pulmonar. Otra forma de compromiso respiratorio es la sinusitis crónica ^2^^,^^8^.

La infección diseminada es la forma clínica más frecuente y se define como la afección de diversos órganos o sistemas, entre los que se destacan el sistema respiratorio y la piel, afectada en el 68 % de los casos ^4^. Sin embargo, también puede alterar las articulaciones, los huesos, el peritoneo, los globos oculares y el sistema nervioso central ^1^. La fiebre es un rasgo típico de esta forma de la enfermedad ^15^. La fungemia es un rasgo distintivo (más frecuente que en la candidiasis) y refleja una forma crítica de la enfermedad ^1^^,^^2^. En la mayoría de los casos se presenta cinco días después de la aparición de las lesiones en piel ^20^.

En los casos aquí presentados, más del 90 % de los hemocultivos fueron positivos. La infección diseminada por *Fusarium* spp. es extremadamente rara en pacientes con trasplante de órgano sólido y con HIV, lo que sugiere que en este grupo de pacientes los neutrófilos y los linfocitos T son claves para delimitar la infección y explica por qué la infección es localizada.

*Fusarium* spp. también produce infección pulmonar en receptores de trasplante de progenitores hematopoyéticos y trasplante hepático ^21^^,^^22^, especialmente en quienes adquieren el hongo por vía aérea ^12^. La fusariosis ocasionalmente se describe en personas inmunocompetentes, como el caso de siete pacientes que presentaron fungemia por *F. verticilloides* debido a la exposición a esporas durante las actividades de construcción en un hospital ^23^.

Las infecciones oculares como queratitis y endoftalmitis, tanto en individuos inmunocompetentes como inmunodeprimidos, se han reportado en personas usuarias de lentes de contacto por el uso de una solución específica tópica contaminada con *Fusarium* spp. ^24^. Uno de los pacientes de la serie presentó compromiso ocular por endoftalmitis aguda unilateral no asociada al uso de lentes de contacto (Figura 1D).

Las manifestaciones del sistema nervioso central en la infección diseminada por *Fusarium* spp. incluyen meningoencefalitis y abscesos cerebrales, pero estos son relativamente raros en comparación con los otros síntomas clínicos en la piel, la región sinonasal y los pulmones ^11^. Cuando se presenta compromiso del sistema nervioso, se atribuye a diseminación hematógena ^11^. En esta serie no se documentó compromiso del sistema nervioso central.

### Diagnóstico diferencial

Uno de los diagnósticos diferenciales más importantes de la fusariosis es la infección invasora por otros hongos filamentosos, especialmente por *Aspergillus* spp. Para diferenciarla de la fusariosis hay varias herramientas clínicas; como lo demostraron Nucci y colaboradores ^17^, la fiebre es más frecuente en la fusariosis (96,2 % versus 63,9 %, p=0,003), al igual que las lesiones cutáneas y la fungemia ^17^. En esta serie se encontraron hallazgos semejantes. Por otro lado, la neumonía (88,9 % versus 50 %, p=0,001) y la sinusitis (63,9 % versus 38,5 %, p=0,048) son más comunes en la aspergilosis ^17^. Cuando hay compromiso pulmonar, es más común ver el signo de halo en la aspergilosis (62,5 % versus 23,1 %, p=0,02) ^17^.

Cuando hay afectación cutánea, el principal diagnóstico diferencial es el ectima ^8^^,^^10^. Incluso, algunos autores han propuesto a *Fusarium* spp. como parte de una lista de agentes patógenos implicados como agentes etiológicos de algunos casos de ectima ^25^.

### Diagnóstico

El diagnóstico de fusariosis requiere correlacionar elementos de la clínica, la histología, la microbiología e, incluso, la epidemiología de la institución. Aunque el diagnóstico definitivo es la identificación del hongo ^8^, esto no siempre se consigue. En estos casos, los factores de riesgo y las manifestaciones clínicas pueden orientar el diagnóstico. Las guías de la *European Society of Clinical Microbiology and Infectious Diseases* (ESCMID) y la *European Confederation of Medical Mycology* (ECMM) recomiendan fuertemente que, entre los estudios para fusariosis, se realice microscopía directa, cultivos, estudio de patología y tomografía computarizada de tórax ^26^.

El diagnóstico de infección por *Fusarium* spp. requiere el cultivo de tejido para una identificación precisa como se ejemplifica en esta serie, ya que el aislamiento del hongo se hizo con mayor frecuencia en hemocultivos (12/13) y en piel (6/13). *Fusarium* spp. aparece en forma de hifas hialinas tabicadas, con ramificación en ángulo agudo, en la tinción de plata metenamina de Gomori. La apariencia de los macroconidios en forma de canoa o de plátano en la tinción de azul de lactofenol (figura 2B), es una característica patognomónica de *Fusarium* spp.

### Cultivo

Una característica de la fusariosis, que la diferencia de las aspergilosis, son los hemocultivos ^14^. Los hemocultivos son positivos en hasta el 60 % (figura 2A) de los pacientes con fusariosis diseminada, en contraste con aquellos con aspergilosis y otras infecciones invasivas por mohos, donde rara vez son positivos ^2^^,^^8^. Los cultivos de piel también son útiles y pueden llevar a un diagnóstico temprano, pues las lesiones cutáneas suelen preceder a los hemocultivos positivos en cinco días ^8^.

*Fusarium* spp. tiene un crecimiento rápido de 3 a 7 días ^2^^,^^15^ en medios como agar papa dextrosa o Sabouraud dextrosa-sacarosa, entre otros. La temperatura ideal de incubación es de 25 a 28 ^o^C y no debe utilizarse cicloheximida porque puede inhibir su síntesis proteica ^15^. Las colonias son blanco-vellosas (figura 2C) con pigmentos que varían según la especie, y por microscopía se observan hifas delgadas y tabicadas con microconidios y macroconidios fusiformes, cuyas características cambian según la especie ^2^.

### Histología

El estudio de patología muestra hifas hialinas y tabicadas que son indistinguibles de *Aspergillus* spp. u otros agentes patológicos de hialohifomicosis ^2^. En la histología también se observa invasión vascular y trombosis, evidencia definitiva de enfermedad fúngica invasiva ^11^^,^^15^, como los hallazgos que se muestran en el caso dos (figura 3B). Aunque la descripción histológica y las coloraciones con ácido peryódico de Schiff y de Gomori muestran el compromiso de la luz de los vasos sanguíneos y las estructuras micóticas, es necesario correlacionar estas deducciones con hallazgos clínicos y paraclínicos, además de estudios microbiológicos para una hacer clasificación definitiva.

### Otros estudios

Entre las herramientas diagnósticas que permiten configurar el diagnóstico de infección fúngica invasora están las pruebas de reacción antígeno-anticuerpo en suero como el galactomanano y el ^1^^,^^3^-β-D-glucano. En la literatura, usualmente se describe que las infecciones por *Fusarium* spp. son positivas para ^1^^,^^3^- β D-glucano, aunque no es una prueba específica y puede ser positiva en infecciones por otros hongos como *Candida* spp., *Aspergillus* spp. y *Trichosporon* spp.

En revisiones recientes sobre la positividad de estas pruebas en infecciones por *Fusarium* spp., la positividad del ^1^^,^^3^- β -D-glucano y del galactomano fue del 36 y el 9 %, respectivamente ^1^. El galactomanano hace parte de la pared de muchas especies de hongos que pueden causar enfermedad en el ser humano y es más frecuente encontrarlo en infecciones invasivas por *Aspergillus* spp. Sin embargo, hay evidencia de que también puede encontrarse en enfermedades causadas por hongos como *Penicillium* spp., *Fusarium* spp., *Paecilomyces* spp. e *Histoplasma capsulatum*^8^. Algunos autores no han encontrado diferencias estadísticamente significativas en la prueba de galactomanano entre la fusariosis y la aspergilosis (positividad de 73,3 % versus 88,6 %, p=0,18) ^14^.

Otras pruebas reportadas son la PCR y la hibridación *in situ,* esta última con valor predictivo positivo del 100 % ^15^. En esta serie, en el paciente 13 el valor del galactomanano sérico en el momento del diagnóstico de la fusariosis fue de 1,3 ng/ml.

### Imágenes diagnósticas

Como el compromiso primario por *Fusarium* spp. se presenta usualmente por la vía aérea superior (inhalación de esporas) o por pérdida de la barrera cutánea, su manifestación clínica incluye cuadros de neumonía, sinusitis e infecciones en catéteres endovenosos y traumas abiertos. De la misma manera, es común el desarrollo de nódulos o masas (únicas o múltiples) con compromiso aleatorio. Entre los hallazgos en la tomografía de tórax se encuentran: enfermedad de espacio aéreo único y unilateral (29 %), enfermedad de espacio aéreo múltiple y bilateral (24 %), lesiones nodulares (15 %) y lesiones cavitarias (9 %) ^4^. La presencia de nódulos con "signo del halo" solo se reporta en el 20 % de los casos ^27^.

### Tratamiento

El tratamiento agresivo temprano es crítico dada la rápida progresión de la infección y las altas tasas de mortalidad, que alcanzan del 50 al 80 % en casos de fungemia ^3^. El tratamiento de la fusariosis implica una combinación de estrategias: la terapia antifúngica, el desbridamiento quirúrgico, el retiro de dispositivos vasculares -cuando sea necesario- y la recuperación de la neutropenia ^3^.

La sensibilidad *in vitro* de *Fusarium* spp. a las equinocandinas varía entre especies ^28^^-^^30^. Varios estudios ^31^^-^^33^ encontraron que tres equinocandinas (caspofungina, micafungina y anidulafungina) fueron inactivas en 10 cepas de *Fusarium* spp. con una concentración inhibitoria mínima de 8 mg/L ^31^. *Fusarium solani,* en particular, es intrínsecamente resistente a las equinocandinas, por lo que su uso no está recomendado en infección fúngica invasora por *Fusarium* spp.

La anfotericina B se considera el tratamiento de primera opción para la fusariosis invasiva, aunque las concentraciones inhibitorias mínimas entre 1 y 4 mg/L son generalmente sugestivas de resistencia.

Una encuesta epidemiológica reciente sobre infecciones invasivas por *Fusarium* spp. en Europa demostró que los azoles (posaconazol, voriconazol e itraconazol) tienen una concentración inhibitoria mínima más baja contra cepas de *F. verticillioides,* mientras que 14 aislamientos de *F. solani* fueron resistentes a los tres azoles probados ^26^.

El *Clinical and Laboratory Standards Institute* propone valores epidemiológicos de corte de los antifúngicos disponibles para el tratamiento de los complejos de *Fusarium* spp. más frecuentemente aislados, pero no para todas las especies ^3^, y no pueden predecir la respuesta clínica al tratamiento.

El tratamiento de la fusariosis invasiva es un reto por las siguientes razones: *Fusarium* spp. es uno de los hongos más resistentes a los medicamentos; su sensibilidad antifúngica tiende a variar entre las diferentes especies de *Fusarium,* por ejemplo, *F. solani* suele ser más resistente a los agentes antifúngicos en comparación con las otras especies. Por otra parte, no hay reportes de estudios controlados, así que el tratamiento no está bien establecido.

La recomendación para la infección fúngica invasora es la combinación de anfotericina B lipídica y voriconazol. Sin embargo, la efectividad de esta combinación se desconoce porque la información disponible es limitada. Hay algunos informes de casos aislados sobre el resultado exitoso con la combinación de anfotericina B y voriconazol, al menos, por 12 semanas y con recuperación inmunológica ^34^.

La terapia antifúngica combinada es bien tolerada con toxicidad menor y teóricamente puede estabilizar la infección y prevenir la progresión fatal ^35^. En una serie de casos de 26 pacientes y en la revisión simultánea de 97 casos reportados en la literatura desde el año 2000 ^23^, se encontró que algunos recibieron monoterapia con anfotericina o voriconazol y en otros se utilizaron terapias combinadas. Al comparar las frecuencias de mortalidad en los pacientes incluidos en la serie con monoterapia (66 %) versus las terapias combinadas (0 %), y los casos registrados de la revisión de la literatura (31 % en terapia combinada versus 10 % en monoterapia) no se puede concluir cuál es la mejor terapia. No obstante, los pacientes en los que se utilizó más de un fármaco se encontraban más críticamente enfermos, pero a la fecha no se dispone de estudios clínicos que apoyen esta observación.

La dosis recomendada de voriconazol en adultos es de 6 mg/kg, vía endovenosa, cada 12 horas como dosis de carga y después una dosis de mantenimiento de 4 mg/kg cada 12 horas ^26^. La dosis diaria recomendada de anfotericina B liposómica es de 5 a 10 mg/kg ^15^. Algunos autores recomiendan adicionar el factor estimulante de colonia de granulocitos-macrófagos, a una dosis diaria de 5 µg/kg, como adyuvante en pacientes neutropénicos ^15^^,^^26^.

La sinergia entre terbinafina y antifúngicos azoles ha sido demostrada en estudios *in vitro.* Sin embargo, se requieren más datos al respecto ^1^. El posaconazol se ha propuesto como terapia de rescate para quienes no responden al tratamiento convencional o presenten intolerancia a la anfotericina B ^36^.

El tratamiento quirúrgico asociado al uso de antifúngicos puede ser necesario. Se ha descrito en informes de caso como un tratamiento agresivo, pero exitoso y como la mejor oportunidad de supervivencia cuando la fusariosis involucra órganos como el pulmón o el hueso y hay compromiso neurológico con riesgo de herniación ^37^^-^^39^. En los casos de las neoplasias hematológicas, la recuperación de la neutropenia es fundamental para el control de la infección fúngica invasora porque disminuye la mortalidad.

En pacientes no neutropénicos, cuya única manifestación sea la fungemia asociada al catéter venoso, suele ser suficiente el retiro del dispositivo ^14^. En esta serie, se retiró el dispositivo vascular central de un paciente, por ser el foco de la infección fúngica invasora y se acompañó con tratamiento antifúngico combinado. Dadas las dificultades en la identificación de la especie de *Fusarium,* la necesidad de un inicio precoz de la terapia antifúngica -en el contexto de pacientes con neoplasias hematológicas y la presencia de neutropenias profundas y prolongadas- se considera que la terapia inicial combinada de voriconazol y anfotericina B es una opción razonable mientras se define la especie de *Fusar um*^40^^-^^42^.

### Prevención

La prevención de la enfermedad diseminada es un aspecto importante del tratamiento, ya que los senos paranasales y el aparato pulmonar son la puerta de entrada más frecuente, seguidos de la zona periungular y los tejidos blandos. Se deben implementar procedimientos de limpieza ambiental para reducir la posibilidad de infección por moho en pacientes inmunocomprometidos, en particular, en aquellos con neutropenia profunda ^12^.

Como la piel y las uñas son fuentes de la infección diseminada, es conveniente hacer un adecuado examen dermatológico antes de someter a un paciente a algún tratamiento inmunosupresor e instruirlo para que evite la manipulación inapropiada de las uñas ^8^.

### Pronóstico

Stempel *etal.* encontraron que la probabilidad de supervivencia a las seis semanas es del 66,7 % y a las 12 semanas es del 53,3 % ^1^. El pronóstico depende del estado inmunológico del huésped ^4^^,^^33^. Los pacientes neutropénicos suelen tener un curso rápido y letal ^4^; la supervivencia de los pacientes con neutropenia persistente es de alrededor del 4 % a pesar de un tratamiento agresivo ^43^. De ahí que la recuperación de la neutropenia sea un factor protector para la supervivencia (razón de probabilidad=0,12; p=0.006) ^4^^,^^7^. Otro factor protector es el compromiso exclusivo de la piel y los tejidos blandos ^4^.

Por otro lado, la hipoalbuminemia, la presencia de fungemia, el desarrollo de la infección fúngica invasora en la unidad de cuidados intensivos o el tratamiento allí después del diagnóstico de la infección fúngica invasora son variables asociadas con la mortalidad ^4^. La mortalidad en esta serie fue mayor del 60 % a pesar del tratamiento antifúngico combinado en los pacientes afectados.

## Conclusión

En los pacientes con cáncer, y en especial en aquellos con neoplasia hematológica, se debe sospechar una infección fúngica invasora cuando a pesar del tratamiento antimicrobiano de amplio espectro persista la neutropenia febril, haya compromiso de piel por la presencia de lesiones polimorfas, de distribución generalizada o de lesiones periungulares, como la paroniquia caracterizada por el compromiso del dedo gordo del pie. En estos pacientes se indica el cultivo de sangre y de biopsia de piel como herramientas diagnósticas para confirmar la fusariosis lo más pronto posible.

Ante la sospecha diagnóstica, es importante iniciar el tratamiento antifúngico basado en el conocimiento epidemiológico de los patrones de sensibilidad. Teniendo en cuenta que *F. solani* es la especie más frecuentemente identificada y que presenta resistencia a todos los azoles, se recomienda la terapia antifúngica combinada con anfotericina B liposómica y voriconazol. La recuperación de la neutropenia es definitiva para el control de la infección fúngica invasora por *Fusarium* spp. ya que puede disminuir la mortalidad.
